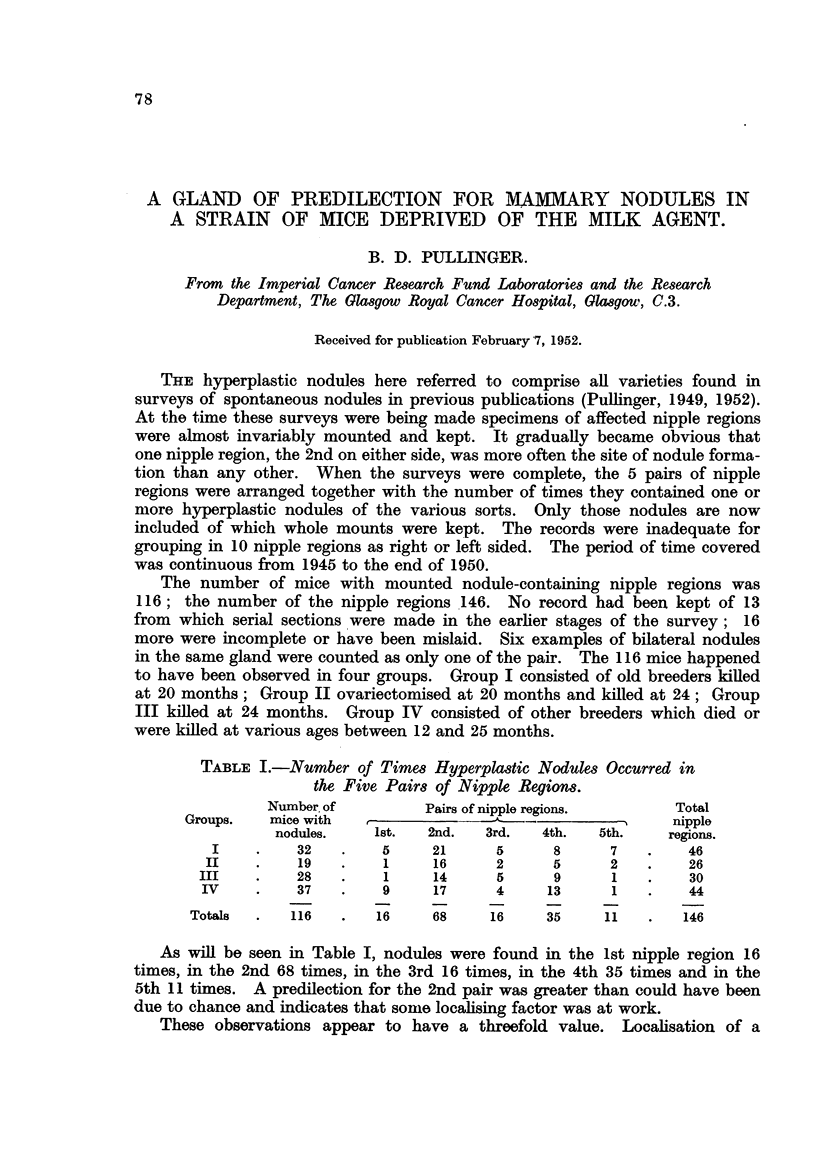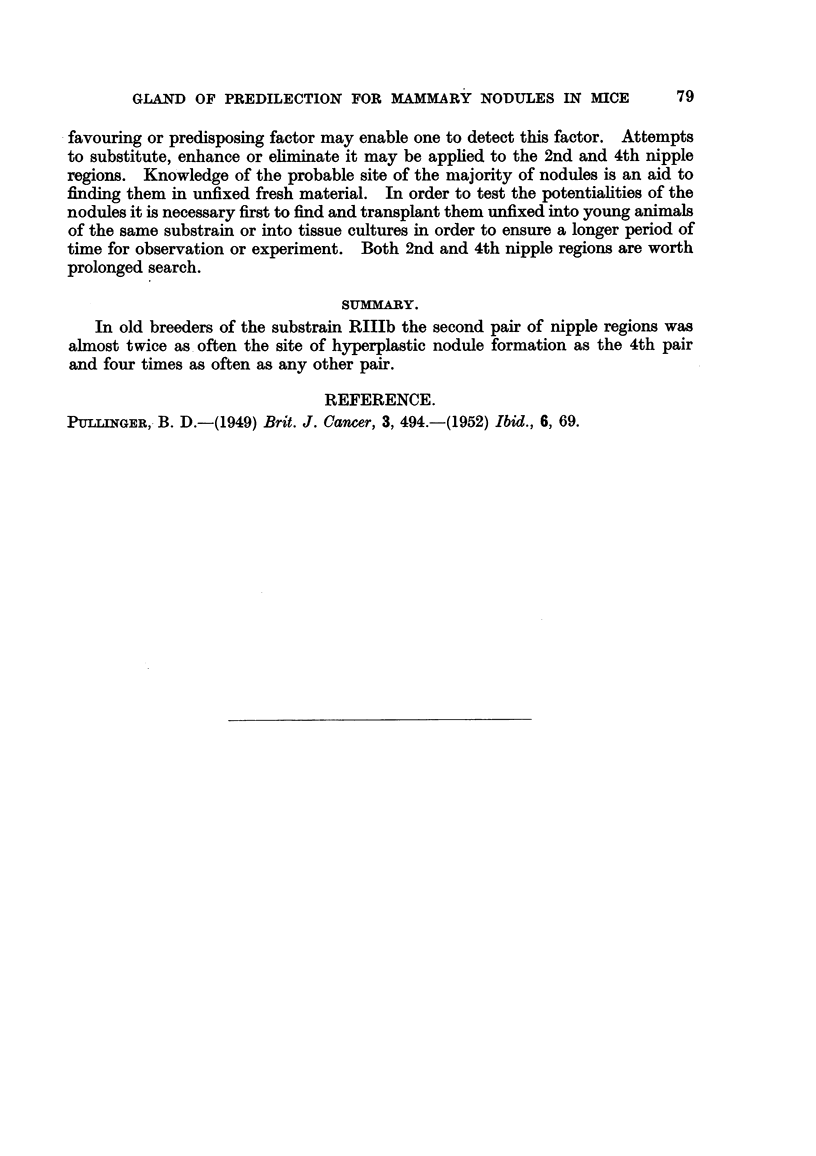# A Gland of Predilection for Mammary Nodules in a Strain of Mice Deprived of the Milk Agent

**DOI:** 10.1038/bjc.1952.7

**Published:** 1952-03

**Authors:** B. D. Pullinger


### A GLAND OF PREDILECTION FOR MAMMARY NODULES IN A STRAIN OF MICE DEPRIVED OF THE MILK AGENT.

B. D. PULLINGER.

*From the Imperial Cancer Research Fund Laboratories and the Research Department, The Glasgow Royal Cancer Hospital, Glasgow, C.3.*

Received for publication February 7, 1952.

THE hyperplastic nodules here referred to comprise all varieties found in surveys of spontaneous nodules in previous publications (Pullinger, 1949, 1952). At the time these surveys were being made specimens of affected nipple regions were almost invariably mounted and kept. It gradually became obvious that one nipple region, the 2nd on either side, was more often the site of nodule formation than any other. When the surveys were complete, the 5 pairs of nipple regions were arranged together with the number of times they contained one or more hyperplastic nodules of the various sorts. Only those nodules are now included of which whole mounts were kept. The records were inadequate for grouping in 10 nipple regions as right or left sided. The period of time covered was continuous from 1945 to the end of 1950.

The number of mice with mounted nodule-containing nipple regions was 116; the number of the nipple regions 146. No record had been kept of 13 from which serial sections were made in the earlier stages of the survey; 16 more were incomplete or have been mislaid. Six examples of bilateral nodules in the same gland were counted as only one of the pair. The 116 mice happened to have been observed in four groups. Group I consisted of old breeders killed at 20 months; Group II ovariectomised at 20 months and killed at 24; Group III killed at 24 months. Group IV consisted of other breeders which died or were killed at various ages between 12 and 25 months.

TABLE I.—*Number of Times Hyperplastic Nodules Occurred in the Five Pairs of Nipple Regions.*

| Groups. | Number of mice with nodules. | Pairs of nipple regions. 1st. | 2nd. | 3rd. | 4th. | 5th. | Total nipple regions. |
|---|---|---|---|---|---|---|---|
| I | 32 | 5 | 21 | 5 | 8 | 7 | 46 |
| II | 19 | 1 | 16 | 2 | 5 | 2 | 26 |
| III | 28 | 1 | 14 | 5 | 9 | 1 | 30 |
| IV | 37 | 9 | 17 | 4 | 13 | 1 | 44 |
| Totals | 116 | 16 | 68 | 16 | 35 | 11 | 146 |

As will be seen in Table I, nodules were found in the 1st nipple region 16 times, in the 2nd 68 times, in the 3rd 16 times, in the 4th 35 times and in the 5th 11 times. A predilection for the 2nd pair was greater than could have been due to chance and indicates that some localising factor was at work.

These observations appear to have a threefold value. Localisation of a favouring or predisposing factor may enable one to detect this factor. Attempts to substitute, enhance or eliminate it may be applied to the 2nd and 4th nipple regions. Knowledge of the probable site of the majority of nodules is an aid to finding them in unfixed fresh material. In order to test the potentialities of the nodules it is necessary first to find and transplant them unfixed into young animals of the same substrain or into tissue cultures in order to ensure a longer period of time for observation or experiment. Both 2nd and 4th nipple regions are worth prolonged search.

### SUMMARY.

In old breeders of the substrain RIIIb the second pair of nipple regions was almost twice as often the site of hyperplastic nodule formation as the 4th pair and four times as often as any other pair.

### REFERENCE.

PULLINGER, B. D.—(1949) *Brit. J. Cancer*, **3**, 494.—(1952) *Ibid.*, **6**, 69.